# *In Vitro* Antioxidant and Antiproliferative Activities of Methanolic Plant Part Extracts of *Theobroma cacao*

**DOI:** 10.3390/molecules191118317

**Published:** 2014-11-10

**Authors:** Zainal Baharum, Abdah Md Akim, Yun Hin Taufiq-Yap, Roslida Abdul Hamid, Rosmin Kasran

**Affiliations:** 1 Department of Biomedical Science, Faculty of Medicine and Health Sciences, University Putra Malaysia, 43400 Serdang, Selangor, Malaysia; E-Mails: abdah@upm.edu.my (A.M.A.); roslida@upm.edu.my (R.A.H.); 2 Division of Biotechnology, Centre for Cocoa Biotechnology Research, Malaysian Cocoa Board, Commercial Zone 1, North KKIP, Norowot Road, 88460 Kota Kinabalu Industrial Park, Sabah, Malaysia; E-Mail: rosmin@koko.gov.my; 3 Department of Chemistry, Faculty of Science, University Putra Malaysia, 43400 Serdang, Selangor, Malaysia; E-Mail: taufiq@upm.edu.my

**Keywords:** *Theobroma cacao*, plant, antiproliferative, antioxidant, phenolics, natural product

## Abstract

The aims of this study were to determine the antioxidant and antiproliferative activity of the following *Theobroma cacao* plant part methanolic extracts: leaf, bark, husk, fermented and unfermented shell, pith, root, and cherelle. Antioxidant activity was determined using 2,2-diphenyl-2-picrylhydrazyl (DPPH), thiobarbituric acid-reactive substances (TBARS), and Folin-Ciocalteu assays; the 3-[4,5-dimethylthiazol-2-yl]-2,5-diphenyltetrazolium (MTT) assay was used to determine antiproliferative activity. The root extract had the highest antioxidant activity; its median effective dose (EC_50_) was 358.3 ± 7.0 µg/mL and total phenolic content was 22.0 ± 1.1 g GAE/100 g extract as compared to the other methanolic plant part extracts. Only the cherelle extract demonstrated 10.4% ± 1.1% inhibition activity in the lipid peroxidation assay. The MTT assay revealed that the leaf extract had the highest antiproliferative activity against MCF-7 cells [median inhibitory concentration (IC_50_) = 41.4 ± 3.3 µg/mL]. Given the overall high IC_50_ for the normal liver cell line WRL-68, this study indicates that *T. cacao* methanolic extracts have a cytotoxic effect in cancer cells, but not in normal cells. Planned future investigations will involve the purification, identification, determination of the mechanisms of action, and molecular assay of *T. cacao* plant extracts.

## 1. Introduction

Cancer is a complex multifactorial cell disease characterized by abnormal cellular proliferation. Cancer development and progression are dependent on the cellular accumulation of various genetic and epigenetic events [1,2], and is an aberrant net accumulation of typical cells arising from excess proliferation, insufficient apoptosis, or a combination thereof [3]. Cancer development is normally caused by oncogene, tumor suppressor gene, and microRNA gene alterations [4]. It imposes a serious burden on the public health system, and its treatment and cure are scientifically challenging. Cancer is expected to claim nine million lives worldwide by 2015 [5]. In Malaysia, cancer is the fourth leading cause of death; it is the second leading cause of death in developed countries after cardiovascular diseases. The Ministry of Health of Malaysia has reported that deaths from breast cancer rank among the top 10 cancer-related deaths in the country. Currently, about 65% of drugs used in chemotherapy are of natural origin [6]. Medicinal plants have a long history in both traditional and modern cancer treatments [7,8] and have been used to treat human diseases for centuries [9,10,11]. Thus, it is possible that traditional medicinal plants can serve as potential sources for developing new drugs and more effective anti-cancer agents for future therapy [12].

*Theobroma cacao* (*T. cacao*) or cocoa, was considered a divine food by the Olmec, Maya, and Aztec civilizations that consumed its products as early as 600 BC. Several documents, among them the Badianus Manuscript, the Florentine Codex, and the Princeton Codex, show over 100 documented medicinal uses for cocoa. More than 200 studies have reported the bioactive compounds, chemical composition, and health benefits of cocoa and cocoa products [13,14,15]. Mhd. Jalil and Ismail [16] reported that various parts of the cocoa plant, e.g., cocoa beans (prepared as chocolate), the bark, flower, pulp, and leaf, and cocoa butter have been used for medicinal purposes. The phenolic compounds in cocoa contain bioactive compounds that have potential health benefits for chronic diseases such as inflammation, cardiovascular illness, neurodegenerative disorders, and cancer [17,18,19]. A naturally occurring cocoa-derived pentameric procyanidin (pentamer) caused G0/G1 arrest by selectively inhibiting the proliferation of human breast cancer cells (MDA-MB-231, MDA-MB-436, MDA-MB-468, SKBR-3, MCF-7) and benzo(a)pyrene-immortalized 184A1N4 and 184B5 cells [20]. The flavonols in cocoa exist as both monomeric flavonols, (−)-epicatechin, and to a much lesser extent, (+)-catechin, and are structurally related oligomers (procyanidins). Cocoa flavonols and procyanidins induced 70% growth inhibition with cell cycle blockade in Caco-2 cells at the G2/M phase [21].

Our objectives were to determine the antioxidant and antiproliferative activity of the methanolic extract of the following cocoa plant parts: leaf, bark, husk, fermented and unfermented shell, pith, root, and cherelle, against the estrogen receptor–positive (MCF-7) and estrogen receptor–negative (MDA-MB-231) breast cancer cell lines; liver (HepG2), colon (HT-29), lung (A549), and cervical (HeLa) cancer cell lines; and a normal liver cell line (WRL-68).

## 2. Results and Discussion

### 2.1. Antioxidant Activity

Free radicals are the primary cause of oxidative damage of biological molecules in the human body, and are related to coronary heart disease, aging, cancer, and dementia [22]. We used the 2,2-diphenyl-2-picrylhydrazyl (DPPH) assay to determine the antioxidant activity of cocoa plant methanolic extracts, *i.e.*, their ability to scavenge free radicals; concentrations of the extract and control enabling the scavenging of 50% of 300 µM DPPH was detected. As shown in Table 1, the median effective concentrations (EC_50_) of the leaf, bark, root, and cherelle extracts were 433.3 ± 22.2, 396.3 ± 0.9, 358.0 ± 7.0, and 390.0 ± 7.0 µg/mL, respectively. However, no EC_50_ values were obtained for the husk, fermented and unfermented shell, and pith extracts, as very low to no antioxidant activity was detected in these extracts. The root extract had the highest antioxidant activity among all the extracts (EC_50_ = 358.0 ± 7.0 µg/mL), which was significant (*p* < 0.05) among the root, leaf, and cherelle extracts, but no significant (*p* > 0.05) with bark extract. A higher DPPH radical scavenging activity is associated with a lower EC_50_, and it was evident that the extracts could donate hydrogen to act as antioxidants [23]. We estimated the radical scavenging activity of the extracts by comparing the percentage scavenging activity of DPPH radical formation by the extracts to that of Trolox (positive control). Trolox had the highest antioxidant activity (EC_50_ = 187.7 ± 29.8 µg/mL, *p* < 0.05) compared to the extracts because it is a pure antioxidant compound (Table 1). We detected varying levels of scavenging activity in the individual extracts, which we believe was due to the different mechanisms involved in the radical–antioxidant reaction [24].

**Table 1:** Antioxidant activity of cocoa plant part extracts.

Plant Part	EC_50_ (µg/mL) *
Leaf	433.3 ± 22.2 ^d^
Bark	396.3 ± 0.9 ^a^
Husk	-
Unfermented shell	-
Fermented shell	-
Pith	-
Root	358.3 ± 7.0 ^a,b^
Cherelle	390.0 ± 7.0 ^b,c^
Positive control (Trolox)	187.7 ± 29.8 ^e^

* Values are the means of three replicate samples (*n* = 3). Data are presented as the mean ± SEM. Superscript letters indicate significant difference according to the Tukey honest significant difference test (*p* < 0.05).

### 2.2. Total Phenolics Content

Phenolic compounds are very important plant constituents because they exert antioxidant activity by inactivating lipid free radicals or by preventing hydroperoxide decomposition into free radicals. In this study, the total phenolic content of cocoa plant part methanolic extracts was measured in units of g gallic acid/100 g extract equivalent of phenolic compound (Table 2). The root extract had the highest total phenolic content (22.0 ± 1.1 g GAE/100 g extract), followed by cherelle (19.6 ± 0.3 g/100 g GAE/100 g extract), bark (15.4 ± 0.1 g GAE/100 g extract), leaf (13.3 ± 0.2 g GAE/100 g extract), husk (8.7 ± 0.4 g GAE/100 g extract), fermented shell (4.3 ± 0.3 g GAE/100 g extract), and unfermented shell (1.5 ± 0.1 g GAE/100 g extract); the pith extract had the lowest total phenolic content (1.2 ± 0.1 g GAE/100 g extract).

**Table 2:** Total phenolic content of cocoa plant part extracts.

Plant Part	Total Phenolic Content * (g GAE/100 g Extract)
Leaf	13.3 ± 0.2 ^b^
Bark	15.4 ± 0.1 ^c^
Husk	8.7 ± 0.4 ^d^
Unfermented shell	1.5 ± 0.1 ^a^
Fermented shell	4.3 ± 0.3 ^e^
Pith	1.2 ± 0.1 ^a^
Root	22.0 ± 1.1 ^f^
Cherelle	19.6 ± 0.3

* Values are means of three replicate samples (*n* = 3). Data are presented as the mean ± SEM. Superscript letters indicate significant difference according to the Tukey honest significant difference test (*p* < 0.05). GAE, gallic acid equivalents.

The total phenolic content determined using the Folin-Ciocalteu method does not involve absolute measurements of the amounts of phenolic compounds, but are based on their chemical reducing capacity relative to gallic acid [25]. Many studies have reported that cocoa phenolics are bioactive compounds with potential health benefits for various chronic diseases, including inflammation, cardiovascular illness, neurodegenerative disorders, and cancer [19], and these activities might be related to their antioxidant activity [26]. Our findings indicate that the total phenolic content differs among different cocoa plant parts.

### 2.3. Lipid Peroxidation Inhibition Activity

*In vitro* lipid peroxidation inhibition activity was evaluated based on the determination of malondialdehyde (MDA) and related compounds in rat liver homogenate [27]. Thiobarbituric acid (TBA) reacts with MDA to form a diadduct, a pink chromogen, which can be detected at 532 nm using spectrophotometry. MDA is one of the major degradation products of lipid peroxidation and is studied widely as an index of lipid peroxidation, serving as an oxidative stress marker [28]. Lipid peroxidation is accelerated when free radicals are formed as a result of losing a hydrogen atom from the double bond in the unsaturated fatty acid structure. Free radical scavenging is a major antioxidation mechanism for inhibiting the lipid peroxidation chain reaction [29]; therefore, inhibiting lipid peroxidation is considered an important index of antioxidant activity. Table 3 describes the lipid peroxidation activity of the cocoa plant part methanolic extracts. The cherelle extract had weak activity, inducing 10.4% ± 1.1% inhibition at the maximum concentration of 10 mg/mL compared to the Trolox standard of 32.6% ± 3.2% inhibition, but no activity was observed for the leaf, bark, husk, unfermented and fermented shell, pith, and root extracts. The results suggest that these extracts had no anti-lipid activity due to the absence of excellent hydroxyl scavenging activity in the compounds in the methanolic extracts, which is significant for diseases caused by oxidative stress.

**Table 3:** Lipid peroxidation activity of cocoa plant part extracts.

Plant Part	Inhibition (%) *
Leaf	-
Bark	-
Husk	-
Unfermented shell	-
Fermented shell	-
Pith	-
Root	-
Cherelle	10.4 ± 1.1
Trolox (Positive control)	32.6 ± 3.2

* Values are means of three replicate samples (*n* = 3). Data are presented as the mean ± SEM.

### 2.4. Antiproliferative Activity

A previous study reported that the *T. cacao* bean is a promising source of anti-cancer chemopreventive agents [30]. In the present study, the anti-cancer effect of methanolic extracts of cocoa plant parts against the human cancer cell lines A549, HeLa, HepG2, HT-29, MCF-7, and MDA-MB-231 were investigated. Based on the report by Atjanasuppat *et al.* the antiproliferative activities of the extracts were categorized according to the median inhibitory concentration (IC_50_) into four groups: ≤20 µg/mL, active; >20–100 µg/mL, moderately active; >100–1000 µg/mL, weakly active; and >1000 µg/mL, inactive [31]. The anti-cancer activities of the extracts were preliminarily screened by MTT assay, the percentage viability curves of treated cells were plotted against the extract concentrations, and the IC_50_ as compared to that of untreated cells was determined. According to the United States National Cancer Institute plant screening program, a crude extract is generally considered to have *in vitro* cytotoxic activity if the IC_50_ is <30–40 µg/mL [32]. MTT screening of the antiproliferative activities of the extracts determined that the following extracts were moderately active (IC_50_ < 100 µg/mL) against human cancer cell lines: leaf (41.4 ± 3.3 µg/mL against MCF-7 cells), bark (72.0 ± 9.3 µg/mL against MCF-7 cells), husk fermented shell (71.4 ± 12.11 and 68.9 ± 10.2 µg/mL against HeLa and HepG2 cells, respectively), root (76.4 ± 13.8 µg/mL against MCF-7 cells), and cherelle (67.8 ± 9.4 and 68.9 ± 11.4 µg/mL against HeLa and MCF-7 cells, respectively). The extracts that were weakly active against cancer cell lines (IC_50_ = 100–1000 µg/mL) are listed in Table 4. The leaf extract had the most potent anti-cancer activity against MCF-7 cells (IC_50_ = 41.4 ± 3.3 µg/mL) compared to the other extracts. Plants play an important role in medicine, and most anti-cancer constituents are from leaf extracts, for example, the leaves of *Curcumin* [33], *Alnus sieboldiana* [34], *Hibiscus sabdariffa* [35], *Azadirachta indica* [36], and *Carpinus betulus* [37]. Based on the IC_50_, the results in Table 4 show that all of the extracts had low activity against WRL-68 cells as compared to their activity against the cancer cells. The IC_50_ of the leaf extract against WRL-68 cells was 765.0 ± 34.0 µg/mL, higher than that against MCF-7 cells, which was 41.4 ± 3.3 µg/mL, suggesting that the cocoa leaf methanolic extract is more toxic against cancer cells than normal cells. Al-Rashidi *et al.* [38] noted that low toxicity towards normal cells and high toxicity towards cancer cells indicates that a plant extract has good anti-cancer constituents, and shows that the plant extract has a cytotoxic effect on cancer cells without causing toxicity in normal cells. The leaf extract was selected for further isolation and purification analysis using bioassay-guided fractionation, chromatography, and spectroscopy.

**Table 4:** Antiproliferative activity of cocoa plant part extracts.

Plant Part	IC_50_ (μg/mL) *						
	A549	HeLa	HepG2	HT-29	MCF-7	MDA-MB-231	WRL-68
Leaf	541.3 ± 69.8	430.7 ± 82.2	493.3 ± 17.2	559.0 ± 16.0	41.4 ± 3.3	504.3 ± 30.2	765.00 ± 34.0
Bark	574.7 ± 48.8	688.7 ± 64.8	828.3 ± 154.0	468.7 ± 22.2	72.0 ± 9.3	555.3 ± 36.2	842.67 ± 66.5
Husk	533.7 ± 63.5	372.7 ± 16.5	396.0 ± 40.4	443.3 ± 23.4	62.2 ± 14.9	550.7 ± 6.7	792.33 ± 75.5
Unfermented shell	613.7 ± 25.9	468.3 ± 36.0	464.3 ± 42.8	163.0 ± 18.6	65.0 ± 4.2	555.0 ± 10.1	97.90 ± 1.3
Fermented shell	520.7 ± 19.1	71.4 ± 12.1	68.9 ± 10.2	197.0 ± 13.2	242.3 ± 47.7	486.3 ± 13.0	633.33 ± 119.8
Pith	595.0 ± 82.6	868.0 ± 13.6	951.0 ± 13.8	530.3 ± 41.1	329.7 ± 52.7	360.0 ± 34.8	489.00 ± 50.6
Root	636.3 ± 66.4	321.7 ± 26.0	237.3 ± 19.0	613.3 ± 60.2	76.4 ± 13.8	400.3 ± 74.6	>1000.0
Cherelle	639.7 ± 45.7	67.8 ± 9.4	427.3 ± 31.4	629.3 ± 37.8	68.9 ± 11.4	614.7 ± 11.3	857.00 ± 44.0

* Values are means of 3 replicate samples (*n* = 3). Data are presented as the mean ± SEM.

### 2.5. Correlation between Anti-Cancer Activity and Antioxidant Activity and Total Phenolic Content

As many plant phenolic compounds are good sources of natural antioxidants [39], we determined the Pearson correlation coefficients between the antioxidant activity and total phenolic content of the extracts (Table 5). There was a strong significant relationship between antioxidant activity and total phenolic content (correlation coefficient, r = ~0.9, *p* < 0.01), indicating that high phenolic content is a significantly important factor for determining the antioxidant activity of cocoa plant part extracts. The antioxidant activity of cocoa co-products may be attributed to the phytochemical compounds they contain, especially the polyphenolic compounds, *i.e.*, mainly flavan-3-ol compounds such as the monomers catechin and epicatechin and the dimer procyanidin B2. In addition to phenolic compounds, the presence of methylxanthine (theobromine and caffeine) and anthocyanins in cocoa beans might influence the antioxidant capacity. Recent studies have also demonstrated that the individual antioxidant activity of phenolic compounds in model systems have mutually synergistic or antagonistic effects [40,41]. In this study, the leaf extract displayed the strongest anti-cancer effect but moderate antioxidant activity and total phenolic content. This suggests that antioxidant activity and total phenolic content have a moderate effect on the anti-cancer activity of cocoa plant part extracts. The statistical analysis supported these results, where the anti-cancer activities of the extracts showed negative moderate correlation with their antioxidant activity (r = −0.6) and total phenolic content (r = −0.6), but not significantly (*p* > 0.01) (Table 5). However, the inhibition of cancer cell proliferation by the extracts may not be wholly due to their polyphenolic content, but might be attributed to other bioactive compounds present.

**Table 5:** Bivariate correlation of anti-cancer activities, antioxidant activity, and total phenolic content of cocoa plant part extracts.

Relationship	Correlation Coefficient	*p* (2-Tailed)
DPPH and total phenolic content	0.872	0.005
DPPH and anti-cancer activity (MCF-7 cells)	−0.563	0.146
Total phenolic content and anti-cancer activity (MCF-7 cells)	−0.592	0.122

**Table 6:** Phytochemical constituents of cocoa plant part extracts.

Compound	Cocoa Plant Part Extracts							
	L	H	P	R	B	SF	SUF	C
Alkaloids	-	-	-	-	-	-	-	-
Saponins	1+	1+	1+	1+	2+	-	1+	1+
Flavonoids	1+	2+	-	-	-	1+	-	1+
Hydrolysable tannins	-	-	-	-	-	-	-	-
Condensed tannins	2+	1+	-	2+	2+	1+	-	2+
Triterpenes	2+	-	-	-	-	-	-	-
Steroids	2+	-	-	-	-	-	-	-

Note: For saponins: 1+, 1–2 cm froth; 2+, 2–3 cm froth; and 3+, >3 cm froth. For flavonoids, tannins, triterpenes, and steroids: 1+, weak color; 2+, mild color; 3+, strong color. For alkaloids: - and 1+, negligible amount of precipitate; 2+, weak precipitate; 3+, strong precipitate. L, leaf; H, husk; P, pith; R, root; B, bark; SF, fermented shell; SUF, unfermented shell; C, cherelle.

### 2.6. Phytochemical Testing

We carried out preliminary phytochemical analysis for the presence of alkaloids, saponins, flavonoids, tannins, polyphenolics, steroids, and triterpenes, known to support bioactive activities in medicinal plants, in the extracts. Overall, saponins, flavonoids, condensed tannins, triterpenes, steroids were detected in the extracts, but not alkaloids or hydrolysable tannins (Table 6). Flavonoids, saponins, triterpenes, and tannins all have antioxidant and anti-cancer activity. The presence of these compounds is believed to contribute partly to their antiproliferative activity through antioxidant and free radical scavenging effects [42,43]. Tannins and polyphenolic compounds are useful for treating inflamed or ulcerated tissues, and have remarkable cancer prevention and anti-cancer activity [44,45]. Thus, cocoa leaf extract, which contained most of the abovementioned phytochemical constituents, may be a potential source of bioactive compounds for treating cancer. The leaf extract also contained steroids, which are very important compounds, especially given their relationship with compounds such as sex hormones. Steroids stimulate menstrual discharge and decrease milk secretion. Flavonoids, also among the leaf extract constituents, have a wide range of biological activities that include antimicrobial, anti-inflammatory, anti-angionic, analgesic, and anti-allergic effects, and cytostatic and antioxidant properties. The ability of flavonoids to scavenge hydroxyl radicals, superoxide anion radicals, and lipid peroxyl radicals, which is important for preventing diseases associated with oxidative damage of membranes, proteins, and DNA [44], highlights many of their health-promoting functions in living organisms. In the human diet, flavonoids may reduce the risk of various cancers as well as prevent menopausal symptoms. Furthermore, the leaf extract contained saponins, which have an inhibitory effect on inflammation, hypocholesterolemia, and diabetes [46]. However, the leaf extract did not contain alkaloids, which have been associated with medicinal uses for centuries, and of which one common bioactivity property is cytotoxicity; their absence in the cocoa plant tends to decrease the risk of poisoning by *T. cacao* [45].

## 3. Experimental

### 3.1. Media, Chemicals, and Reagents

Thiazolyl blue tetrazolium bromide (MTT), (±)-6-hydroxy-2,5,7,8-tetramethylchroman-2-carboxylic acid (Trolox), Dulbecco’s modified Eagle’s medium (DMEM), DPPH, dimethyl sulfoxide (DMSO), Folin-Ciocalteu phenol reagent, gallic acid, trypan blue (0.4%), ascorbic acid, TBA, ferric chloride (FeCl_3_), Tris-HCl, sodium carbonate anhydrous, and phosphate-buffered saline (PBS) were purchased from Sigma-Aldrich (St. Louis, MO, USA). Roswell Park Media Institute (RPMI) 1640, penicillin/streptomycin (100×), trypsin-EDTA (1×), and fetal bovine serum mycoplex (FBS) were obtained from PAA Laboratories, GmbH (Haidmannweg, Germany). The methanol (MeOH), *n*-butanol, and chloroform used were of the highest purity (Fisher Scientific, Loughborough, UK).

### 3.2. Collection and Preparation of Plant Materials

Fresh cocoa plant parts (leaf, bark, husk, unfermented and fermented shell, pith, root, cherelle) were collected from a cocoa smallholder field in Ranau, Sabah, Malaysia. Samples were collected during peak cocoa fruiting season in April and November. The cocoa plant parts were harvested, rinsed under tap water, air-dried, and then oven-dried at 40 °C for 5 days. Then, the samples were ground using a commercial blender. Each powdered plant part (5 g) was extracted by soaking in 200 mL MeOH for 3 days at room temperature. The mixture was then filtered using a clean muslin cloth, followed by filter paper. The filtrate was then evaporated to dryness using a rotary evaporator attached to a vacuum pump. The extract was stored at 2–8 °C until used. To test the biological activity, dried crude extracts were dissolved in DMSO, and the stock solutions were later mixed with culture media (DMEM or RPMI 1640) to achieve concentrations of 1000, 100, 10, 1, 0.1, 0.01, and 0.001 µg/mL using 10-fold serial dilutions.

### 3.3. Cell Lines and Cell Culture Preparation

The cell lines used in this study were estrogen receptor–positive (MCF-7) and estrogen receptor–negative (MDA-MB-231) breast cancer cells; liver (HepG2), colon carcinoma (HT-29), lung (A549), and cervical (HeLa) cancer cells; and normal human liver WRL-68 cells. MCF-7, HT-29, MDA-MB-231, and WRL-68 cells were cultured in 89% DMEM with 10% FBS and 1% penicillin/streptomycin. HepG2, HeLa, and A549 cells were cultured in 89% RPMI 1640 with 10% FBS and 1% penicillin/streptomycin. All cells were cultured at 37 °C at 95% humidity and 5% CO_2_ for 3 days until 80%–90% confluent. Subsequently, the spent medium was removed and replaced with fresh medium and incubated again for 24 h. The cell cultures were then washed with PBS 1–2 times and were suspended using trypsin-EDTA. Fresh medium was added to the cells.

### 3.4. Determination of Antioxidant Activity

The antioxidant activity of the extracts was assessed based on the radical scavenging effect of the stable DPPH free radical based on the method of Tyagi *et al.* [47] and using 300 μM DPPH in MeOH. The extracts were dissolved in MeOH, and each extract solution (10 μL) was allowed to react with 200 μL DPPH at 37 °C for 30 min in a 96-well microtiter plate. After incubation, the decrease in absorbance (optical density, OD) of each solution was measured at 490 nm using a microplate reader. Trolox was used as the positive control. For each sample concentration tested, the percentage of DPPH was calculated using the following formula:

Antioxidant activity (%) = (ODcontrol − ODsample)/ODcontrol × 100%
(1)

where ODsample is the OD of the samples or positive control, and ODcontrol is the negative control OD. The values obtained were plotted against the sample concentration to determine the amount of antioxidant necessary to decrease the initial DPPH concentration by 50% (EC_50_). The antioxidant reaction kinetics in the presence of the extracts were compared with that of the reference Trolox. The assay was performed in triplicate.

### 3.5. Determination of Total Phenolic Content

The total phenolic content was determined according to the method of Ismail *et al.* [48] using the Folin-Ciocalteu reagent. Extracts (100 µL, 1 mg/mL) were transferred to a test tube and 0.75 mL Folin-Ciocalteu reagent (previously diluted 10-fold with deionized water) was added and mixed. The mixture was allowed to stand at room temperature for 5 min. Sodium carbonate [0.75 mL, 6% (w/v)] was added to the mixture and mixed gently. After standing at room temperature for 90 min, the absorbance was read at 725 nm using a UV–Vis spectrophotometer. A standard calibration (50–3000 mg/mL) curve was plotted using gallic acid. The total phenolic content was expressed as gallic acid equivalents (mg) per 100 g vegetable extract.

### 3.6. Determination of Lipid Peroxidation Inhibition Activity

The lipid peroxidation inhibition effect of cocoa plant parts was studied *in vitro* using the method of Sreelekshmi *et al.* [49] with some modifications. Rat liver tissue (2.0 g) was sliced and homogenized with 10 mL 15 mM KCl–Tris-HCl buffer (pH 7.2). The reaction solution (3 replications) comprised 0.25 mL liver homogenate, 0.1 mL Tris-HCl buffer (pH 7.2), 0.05 mL 1 mM ascorbic acid, 0.05 mL 4 mM FeCl_3_, and 0.05 mL extract (1–0.156 mg/mL) in each tube. The reaction tube was capped and incubated at 37 °C for 1 h. Then, 0.5 mL 0.1 N HCl, 0.2 mL 9.8% sodium dodecyl sulfate, 0.9 mL distilled water, and 2 mL 0.6% TBA were added to each tube, which was vigorously shaken and placed in a boiling water bath at 100 °C for 30 min. After cooling, the flocculent precipitate was removed by adding 5 mL *n*-butanol, mixed well, and centrifuged at 9,000 rpm for 10 min. The absorbance (Abs) of the supernatant was measured at 532 nm and the percentage of lipid peroxidation inhibition was determined as follows:

Lipid peroxidation inhibition (%) = (Abs control − Abs sample)/Abs control × 100%
(2)

### 3.7. Determination of Antiproliferative Activity

The antiproliferative activity of the extracts against the human cancer cell lines was tested using the microtitration colorimetric method of MTT reduction [50] with minor modification. MTT is used to determine cell viability in cell proliferation and cytotoxicity assays. Exponential-phase cells that were 80%–90% confluent were harvested from maintenance cultures and counted using a hemocytometer with trypan blue solution. Cell suspensions (100 μL) were dispensed in triplicate in 96-well culture plates at optimized concentrations of ~1.0 × 10^5^ cells/mL per cancer cell line. After 24-h incubation at 37 °C, 100 μL culture medium was removed from the wells and 100 μL fresh medium containing the extracts (1000, 100, 10, 1, 0.1, 0.01, 0.001 µg/mL) was added to each well and incubated for another 48 h. Wells containing DMEM or RPMI 1640 were used as the negative controls. At the end of the treatment period, the medium in each well was aspirated and replaced with 20 μL of 5 mg MTT working solution (MTT stock solution mixed with medium to attain a final concentration of 0.5 mg/mL).

Briefly, MTT powder was dissolved in PBS to form an MTT stock solution (5 mg/mL). The stock solution was filter-sterilized through a 0.22 μm filter and stored at −20 °C until used. The cells were incubated at 37 °C for 4 h, and then the medium was aspirated and replaced with 100 μL DMSO to dissolve the formazan crystals formed. The culture plates were shaken for 5 min and the absorbance (OD) of each well was read using an enzyme-linked immunosorbent assay reader at 570 nm (reference wavelength = 630 nm). The percentage of antiproliferative activity compared to that of untreated cells was determined as follows:

Cell viability (%) = (ODsample − ODblank)/(ODcontrol − ODblank) × 100%
(3)

where ODsample is the absorbance of the samples, ODblank the absorbance of the blank (of the respective concentration solutions), and ODcontrol the absorbance of the control wells. The results were produced from three independent experiments, and each experiment was performed in triplicate. The relative viability of the treated cells compared to that of the control cells is expressed as % cell viability.

### 3.8. Phytochemical Analysis

Preliminary phytochemical analysis of the plant extracts was performed using standard procedures. About 5.0 g dried powdered material was analyzed for the presence of phytochemical constituents that usually have biological activities, *i.e.*, alkaloids, flavonoids, saponins, tannins, triterpenes, and steroids. For alkaloid testing, samples were extracted with chloroform, followed by the addition of ammoniacal chloroform. The mixture was then treated with 10% sulphuric acid and tested with Mayer’s reagent. The formation of white precipitate indicated the presence of alkaloids. For flavonoid testing, samples were extracted with chloroform, dissolved in ether, and shaken in 10% ammonia solution. The formation of a yellow color in the ammonia layer indicated the presence of flavonoids. For saponin testing, samples were extracted in MeOH and mixed with distilled water in a test tube. The formation of a stable froth persisting for at least 15 min indicated the presence of saponins. For tannin testing, samples were extracted in MeOH and mixed with 1% FeCl_3_ solution. The formation of a blue-black color indicated the presence of hydrolysable tannins, while a brownish-green color indicated condensed tannins. For triterpene and steroid testing, samples were extracted with chloroform and were tested using the Liebermann-Burchard reagent. The formation of a reddish color indicated the presence of triterpenes; a greenish color indicated steroids. All samples were analyzed at the Laboratory of Phytomedicine, Forest Research Institute of Malaysia, Kepong, Malaysia.

### 3.9. Statistical Analysis

All data are expressed as the mean ± SEM. The free radical scavenging activity values and total phenolic content of the samples were analyzed using 1-way analysis of variance with SPSS version 16.0-2007 software (IBM Corporation, Armonk, NY, USA) using the Tukey honest significant difference test to determine the difference between samples and controls. The MTT assays for each sample were carried out in triplicate on 3 different days. The IC_50_ values were calculated using GraphPad Prism version 5.02-2008 software (GraphPad Software, Inc., La Jolla, CA, USA). Pearson’s correlation coefficient test was used to assess correlations between means. Differences were considered significant at *p* < 0.05.

## 4. Conclusions

We successfully screened the antioxidant and antiproliferative activities of *T. cacao* leaf, bark, husk, unfermented and fermented shell, pith, root, and cherelle methanolic extracts. The root extract has the highest antioxidant activity and total phenolic content compared to the other extracts. However, only the cherelle extract inhibits lipid peroxidation activity. The leaf extract appears to have high potential antiproliferative effects. Linear regression analysis revealed a positive strong correlation between antioxidant activity and total phenolic content, but negative moderate correlation between antioxidant activity and total phenolic content and anti-cancer effect. Future studies will evaluate and investigate the action of *T. cacao* leaf extract against MCF-7 breast cancer cells in greater detail and identify the possible bioactive compounds that contribute to the anti-cancer activity of the extract.
